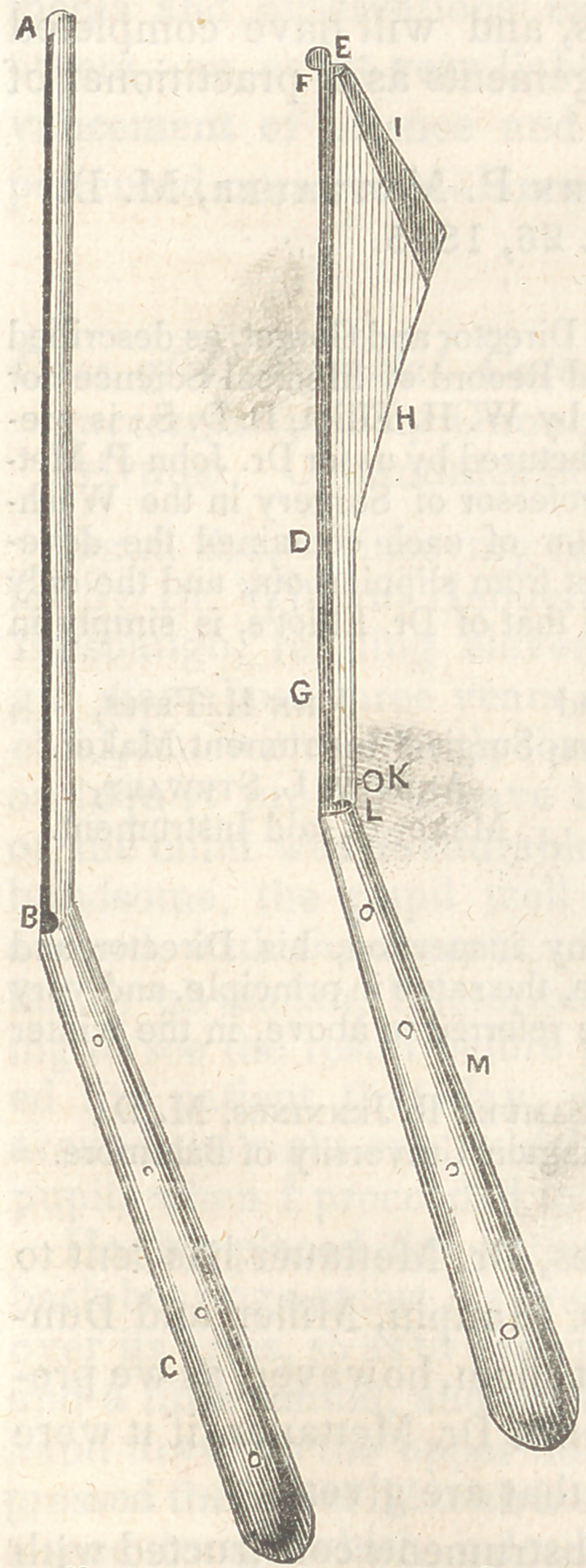# Case of Twins, in Which One Child Was Black, and the Other White

**Published:** 1845-11

**Authors:** Alexander Cunningham


### MEDICAL EXAMINER,

AND

### RECORD OF MEDICAL SCIENCE.

NEW SERIES—No. XI.—NOVEMBER, 1845.

### ORIGINAL COMMUNICATIONS.

*Case of Twins, in which one child was black, and the other white.* By ALEXANDER CUNNINGHAM, M. D. Communicated in a letter to Prof. Dunglison.

*My dear Sir*,—Since my return from Philadelphia last spring, an occurrence of extreme novelty and interest has fallen under my notice, which may be useful to the profession in a physiological point of view. A negro woman, owned by a planter in this neighborhood, aged about 45, after having given birth to thirteen children during her life, none of which were twins, was during the last spring safely delivered of two at one birth, one being *black*, and the other *white*. I saw them when they were a few weeks old, and the contrast in colour, hair, &c., was indeed striking, so much so, that four-fifths of those who examined them were of opinion that the negro was not the mother of both, that some deception was being played; but the mother persisted and still declares them to be her own. That a black woman may give birth, at one time, to a *black* child and a *mulatto*, although extremely rare, I believe ; but a case like the present is a phenomenon as inexplicable as it is interesting. Can you not throw some light upon it? A particular favour would be conferred upon an old pupil, who has heard with delight many of your valuable lessons of instruction.

*Lunenburg County, Virginia*, Sept. 30, 1845.

We think it probable, that there is some mistake in the account of the above case, and that the child said to be white is either an Albino or a mulatto. We hope Dr. Cunningham will investigate the matter further, and give us a more circumstantial account, which we shall be happy to publish.—ED.

*On a new form of Director and Gorget.* By JOHN P. METTAUER, M. D., late Professor of Surgery and Surgical Anatomy in Washington University of Baltimore; now Senior Professor of Prince Edward Medical Institute of Virginia.

In the July No. of the Medical Examiner and Record of Medical Science, under the head of Original Communications, a paper appears entitled "Contributions to General Surgery," by W. H. Elliot, D. D. S. In this paper the writer describes and figures a cutting gorget and staff (director) which he seems to think possesses advantages over the instruments generally employed for lithotomy, in making the section of the urethra and prostate into the bladder; and it would seem, too, that he considers the principles of these improvements original with himself, as no reference is made in the paper to any similar contrivance proposed or employed by any other writer or operative surgeon.

With the principles of these instruments I have been familiar for many years,—since 1818, when the improvement first suggested itself to my mind. I first employed instruments constructed on this plan in operating for stone of the bladder, in 1836, and since then, I have used no others. I have improved upon the original contrivance, and my last improvement was effected during the winter of 1835–36, but the original design of the dove-tail groove of the director, and the globular beak of the gorget have uniformly been adhered to, as it was upon these that the great value of the improvement depended. Being early aware of the danger likely to arise from the accidental escape of the beak of the gorget—or knife, if that instrument be employed—from the groove of the director during its passage into the bladder, especially with inexperienced operators, the dove-tail groove of the director and globular beak of the gorget suggested themselves to my mind as improvements which would prevent such an accident; and abundant experience with the instruments thus modified, conclusively establishes their value and utility in guarding against it in the most effectual manner. Simply inspecting the instruments, if they are properly constructed, would satisfy even the most casual observer, that it would be impossible for the gorget to pursue any other direction than that of the groove of the director. The beak should be well formed with a globular finish, supported by a peduncle of sufficient strength to guard against its being broken by any moderate degree of violence, from a twist, or irregular movement along the groove of the director. The director should be fully six inches long in the shank, with a handle some three or four, and of a size to fill the hand, so that it can be firmly grasped and securely held; and the two branches must form a very obtuse angle, with the groove on the side of the vertex or point of the angle. The director and gorget I employ are delicately formed, and in my opinion should always be so constructed. A probe-point should always form the termination of the director, so as to close that extremity of the groove.

My mode of operating is simply to form the perineo-urethral incision, directed by a staff carried into the bladder; and then to introduce the straight director, already briefly described, through the incision fairly into the bladder, along the groove of which the gorget glides to form the second, or the urethro-prostatic section. As soon as the straight director enters the urethra beyond the proximal angle of the first incision, the staff should be carefully removed from the urethra.

The accompanying sketches will represent the forms of the director and gorget, and will also indicate the dove-tail form of the groove of the director, and the shape and position of the beak of the gorget of half their proper size.

Fig. I represents the dove-tail grooved director (half proper size.) A, probe-point. B, dove-tail groove. C, handle.

Fig. II. D, beak of gorget of half proper size. E, beak made globular. F, neck of beak. G, shank. H, blade. I, cutting-edge. K, screw. L, connection of blade with handle. M, handle. It will be seen that the principles of the director and gorget here described, are identical with those of Dr. Elliot's, and there is no great dissimilarity in form. My instrument was exhibited and described to the medical class during the winter of 1835—6, in the Washington University of Baltimore, while I was connected with that school as Professor of Surgery, and is well remembered by many of the gentlemen now engaged in practice, who honored me with their attendance on my lectures during that period, as well as my private students before that time, and subsequently, who received instruction from me at my present residence in Virginia.

The accompanying certificates will fully sustain my claim to originality and priority over Dr. Elliot, and they are furnished by gentlemen of character and intelligence, and if required, I could procure testimony to establish my claim to the invention as far back as 1818.

Contemplating, and at different times successively effecting modifications in these instruments, and not having brought them to a state of satisfactory perfection until comparatively recently, I have never published an account of them. And such a publication has been still farther delayed by the unfinished state of a work on "the Theory and Practice of Surgery," which I am at this time preparing for the press, and will have completed during the ensuing year, if my engagements as a practitioner of medicine and surgery will permit.

JOHN P. METTAUER, M. D.

*Prince Edward C. H., Va.,* Sept. 26, 1845.

*Sept.* 14, 1845.—This is to certify that the Director and Gorget, as described and figured in the Medical Examiner and Record of Medical Science for July, 1845, for the Operation of Lithotomy, by W. H. Elliot, D. D. S., is precisely the same in principle as those manufactured by us for Dr. John P. Mettauer, as early as 1835–6, when he was Professor of Surgery in the Washington University of Baltimore. The director of each contained the dove-tail groove, to prevent the beak of the gorget from slipping out, and the only difference we see in those made by us and that of Dr. Elliot's, is simply in the handle and the curve of the director.

Signed JOHN H. FRIES,
Foreman for Samuel Jackson, Surgical Instrument Maker.
ANDREW L. STEWART,
Maker of said Instrument.

Test—Samuel Jackson.

Doctor John P. Mettauer submitted to my inspection, his Director and Gorget, constructed with the dove-tail groove, the same in principle, and very nearly the same in every respect, as the one referred to above, in the winter of 1835-6.

SAMUEL R. JENNINGS, M. D.,
Late Professor of Obstetrics, &c., in Washington University of Baltimore.

In addition to the above certificates, Dr. Mettauer has sent to us similar testimonials, signed by Drs. Maupin, Miller and Dunbar; we deem it unnecessary to insert them, however, as we presume no one will doubt the statement of Dr. Mettauer, if it were even unsupported by the certificates that are given.

While we entertain no doubt that instruments constructed with reference to the same mechanical principles as those of Dr. Elliot, were used by Dr. Mettauer as long ago as 1835, we can see no reason for believing that the same plan was not also original with Dr. Elliot. It affords another example of two ingenious men adopting, without concert, the same means to accomplish a particular end. The communication of Dr. Elliot came to us through a friend attached to the Medical Staff of the U. S. Army; one who is well qualified to judge of such an improvement, and who undoubtedly believed the instrument to be original with Dr. Elliot, a gentleman who is represented to be more than ordinarily endowed with mechanical ingenuity. Dr. Mettauer we hope will see the propriety of hereafter publishing his improvements and observations early, to avoid being anticipated by others—an event very liable to happen in this age of rapid advancement of science and the arts, as we have repeatedly experienced ourselves.—Editor.

## Figures and Tables

**Figure f1:**